# Bidirectional alteration of Cav-1 expression is associated with mitogenic conversion of its function in gastric tumor progression

**DOI:** 10.1186/s12885-017-3770-y

**Published:** 2017-11-15

**Authors:** Byung-Kyu Ryu, Min-Goo Lee, Nam-Hoon Kim, Kil Yeon Lee, Shin-Ju Oh, Jung-Rock Moon, Hyo Jong Kim, Sung-Gil Chi

**Affiliations:** 1 School of Life Sciences and Biotechnology, Korea University, Seoul, South Korea; 2 Department of Internal Medicine, College of Medicine, Inje University, Goyang, South Korea; 3 Department of General Surgery, School of Medicine, Kyung Hee University, Seoul, South Korea; 4 Department of Internal Medicine, School of Medicine, Kyung Hee University, Seoul, South Korea

**Keywords:** Caveolin, Promoter methylation, Gastric cancer, RAF, Erk

## Abstract

**Background:**

Expression of caveolin-1 (Cav-1) is frequently altered in many human cancers and both tumor suppression and promotion functions of Cav-1 have been suggested based on its expression status. However, it remains unanswered how Cav-1 provokes opposite effects in different cancers or different phases of tumor progression.

**Methods:**

To explore the implication of Cav-1 alteration in gastric tumorigenesis, the expression and mutational status of Cav-1 and its effects on tumor cell growth were characterized.

**Results:**

A substantial fraction of primary tumors and cell lines displayed abnormally low or high *Cav-1* mRNA expression, indicating the bidirectional alteration of Cav-1 in gastric cancers. While allelic imbalance and mutational alterations of the *Cav-1* gene were rarely detected, aberrant promoter hyper- or hypo-methylation showed a tight correlation with bidirectional alteration of its expression. Abnormally low and high Cav-1 expression was more frequently observed in early and advanced cancers, respectively, suggesting the oncogenic switch of its function in tumor progression. Cell cycle progression, DNA synthesis, and colony forming ability were markedly decreased by Cav-1 transfection in low-expressing tumor cells but by its depletion in high-expressing cells. Interestingly, Cav-1 exerted opposite effects on MEK-ERK signaling in these two cell types through the reciprocal regulation of the RAF-ERK negative feedback loop. A feedback inhibition of RAF by ERK was stimulated by restoration of Cav-1 expression in low-expressing cells but by it depletion in high-expressing cells. As predicted, the opposite effects of Cav-1 on both tumor cell growth and inhibitory RAF phosphorylation were abolished if ERK is depleted.

**Conclusion:**

Bidirectional alteration of Cav-1 is linked to its opposite effects on gastric tumor cell growth, which stem from the reciprocal control on the RAF-ERK negative feedback loop.

## Background

Caveolae are flask-shaped vesicular invaginations of the plasma membrane characterized by the existence of integral membrane proteins termed caveolins. Caveolae is implicated in many cellular functions, including membrane trafficking, endocytosis, lipid metabolism, cell adhesion, signal transduction in cellular proliferation and apoptosis [1]. Caveolins are a family of proteins composed of three isoforms, Caveolin (Cav)-1, −2, and −3. Among the three caveolins, Cav-1 is a principal structural component of caveolae and forms a high molecular complex of homo-oligomer or hetero-oligomer with Cav-2. A scaffolding domain within Cav-1 allows this protein to interact with signaling molecules, including growth factor receptors, G-protein coupled receptors, small GTPases, Src kinases, nitric oxide synthases, and integrins [2]. Integrations and complex formation of Cav-1 with signaling molecules functionally affect the activity of these molecules.

Despite a growing body of evidence on Cav-1 implication in tumorigenesis, its role in tumor growth and underlying molecular mechanisms remain largely undefined. Both tumor suppression and promotion roles of Cav-1 have been proposed on the basis of its expression status detected in cancers. Cav-1 expression is frequently down-regulated in many human cancers mainly due to promoter hypermethylation whereas its elevation correlates with enhanced progression, multidrug resistance, and metastatic potentials of certain tumors [3–6]. Furthermore, *Cav-1* gene amplification and mutation were reported in a subgroup of breast cancers [7, 8]. These findings demonstrate that Cav-1 has differential functions in tumorigenesis depending on the types, origins, or genetic contexts of tumors.

Caveolae have been proposed to be the site of epidermal growth factor receptor (EGFR) signaling, including EGFR autophosphorylation [9]. EGF-induced tumor cell proliferation and migration is suppressed when Cav-1 binds to the EGFR, suggesting that Cav-1 may play a role in maintaining the EGFR in an inactive state, with dissociation from Cav-1 promoting EGFR activation [10]. It was also shown that many components of Ras signaling, including RAF, MEK, and ERK appear to be compartmentalized within caveolin-rich membrane domains and that Cav-1 downregulation results in constitutive activation of ERK signaling while activation of Ras-ERK signaling causes Cav-1 reduction [11, 12]. In contrast, Cav-1 appears to promote metastasis of Ewing sarcoma and the proliferation of metastatic lung cancer cells through activation of the MAPK-ERK pathway [13, 14]. A recent study also showed that Cav-1 is required for kinase suppressor of Ras 1 (KSR1)-mediated ERK1/2 activation, Ras-induced senescence, and transformation [15]. These findings thus indicate that Cav-1 functions as an endogenous inhibitor or stimulator of the Ras-ERK cascade. Nevertheless, the molecular basis for the opposite effects of Cav-1 on EGFR and Ras-MAPK signaling and its implication in tumorigenesis remains largely undefined.

Gastric cancer is one of the most commonly diagnosed malignancies worldwide and a leading cause of cancer mortality in certain areas such as Korea, Japan, South America, and Eastern Europe [16, 17]. Although a number of study indicates that genetic and/or epigenetic alterations of multiple genes, such as *p53*, *K-Ras*, and *E-Cadherin* are associated with the development and progression of gastric cancers, molecular events that drive the neoplastic process remain to be characterized [18]. In this study, we found that *Cav-1* is abnormally down- and up-regulated in a considerable fraction of gastric cancers due to promoter hyper- and hypo-methylation, respectively. In low- and high-expressing tumor cells, Cav-1 evokes the opposite effects on cell proliferation and colony formation through the reciprocal control on the RAF-ERK negative feedback loop. Therefore, our study demonstrates that Cav-1 acts as a positive or negative regulator of the RAF-ERK feedback loop and that the mitogenic switch of Cav-1 function is highly associated with bidirectional alteration of its expression in tumor progression.

## Methods

### Tissues specimens and cell lines

Total 180 gastric tissues including 100 primary carcinomas, 4 adenomas, 6 hamartomas, 6 hyperplastic polyps, and 64 normal gastric tissues were obtained were obtained from 100 gastric cancer patients and 80 noncancer patients by surgical resection in the Kyung Hee University Medical Center (Seoul, Korea). Signed informed consent was obtained from each patient. Tissue specimens were snap-frozen in liquid N_2_ and stored at −70 °C until used. Tissue slices were subjected to histopathological review and tumor specimens composed of at least 70% carcinoma cells and adjacent tissues found not to contain tumor cells were chosen for molecular analysis. Fourteen human gastric cancer cell lines (SNU5, SNU16, SNU216, SNU484, SNU601, SNU620, SNU638, SNU719, MKN1, MKN28, MKN45, MKN74, AGS, and KATO-III) were obtained from Korea Cell Line Bank (Seoul, Korea) or American Type Culture Collection (Rockville, MD).

### Quantitative RT- and genomic PCR

RNA extraction and cDNA synthesis were performed as described previously [19]. Briefly, 1 μg of total cellular RNA was converted to cDNA using random hexamer primers and M-MLV reverse transcriptase (Invitrogen Corporation, Carlsbad, CA). PCR was initially carried out over 24–40 cycles and 12.5 ng cDNA (50 μl PCR reaction) undergoing 30–36 cycles showed logarithmic amplification with primers Cav-1S/Cav-1AS for *Cav-1*, C1αA/C1αAS for *Cav-1α*, C1β/C1βAS for *Cav-1β*, Cav-2S/Cav-2AS for *Cav-2*, C2αA/C2αAS for *Cav-2α*, C2βA/C2βAS for *Cav-2β*, and G2/G3 for an endogenous expression standard gene *GAPDH* (Table 1). PCR was done in 1.5 mM MgCl_2_-containing reaction buffer (PCR buffer II) (Perkin Elmer, Branchburg, NJ) and 10 μl of PCR products were resolved on 2% agarose gels. Quantitation was achieved by densitometric scanning of the ethidium bromide-stained gels. Integration and analysis was performed using Molecular Analyst software program (Bio-Rad, Richmond, CA). For genomic PCR, intron 2 regions of *Cav-1* and *Cav-2* and intron 5 region of *GAPDH* were amplified with intron-specific primers RF2S/RF2AS and G3/G5, respectively (Table 1). Quantitative PCR was repeated at least three times for each specimen and the mean was obtained.

**Table 1:** Primers used for PCR, LOH and bisulfite sequencing analysis

Gene	Primer	Sequence (5′ to 3′)
Cav1	Cav-1S	TCTGGGGCGTCGTGCGCAAA
	Cav-1AS	GAACCTTGATGAAGCCTGTG
	C1⍺A	AGTTTTCATCCAGCCACGGG
	C1⍺AS	TCTTGACCACGTCATCGTTGAG
	C1βA	CATTTTTCCTCCCACCGCCGTT
	C1βAS	AAAACTGTGTGTCCCTTCTG
	RF2S	ATGTATATGTACATCAGGGA
	RF2AS	CAGGCACATAGCTGGGTACC
	SNP-1	GGCTCAACATTGTGTTCCCATTTCAGC
	SNP-2	GTGTCAGGAAGACTGGAAGAGGCA
	P1	TGTGTATTTTGTAAATATGGTATA
	P2	AAGTTAAAGATTTTTATTTTTTATT
Cav2	Cav-2S	ATCTGCAGCCATGCCCTCTTTG
	Cav-2AS	GGGTCCAAGTATTCAATCCTGG
	C2⍺A	ATGGGGCTGGAGACGGAGAA
	C2⍺AS	ACTGAAGGCAGAACCATTAGGCA
	C2βA	TGCGTCCTGTCTCCTCAGCT
	C2βAS	ACTGAAGGCAGAACCATTAGGCA
GAPDH	G2	CATGTGGGCCATGAGGTCCACCAC
	G3	AACCATGAGAAGTATGACAACAGC
	G5	GAGTCCTTCCACGATACCAAAG

### Loss of heterozygosity (LOH) analysis

LOH of the *Cav-1* gene was determined using an intraexonic SNP (5′-AGCATC*C*/*T*-3′) located at +2061 nucleotide (exon 3) from the transcription start site. PCR was performed on each tumor and normal DNA sample pair obtained from 50 patients using primers SNP-1/SNP-2 (Table 1). Five μl of the PCR products were used for cutting with the endonuclease *BtsC*I (NEB, Beverly, MA) and enzyme-digested PCR products were electrophoresed on 2% agarose gels. Signal intensity of fragments and the relative ratio of tumor and normal allele intensities were determined by scanning densitometry.

### RT-PCR-single strand conformation polymorphism (SSCP) analysis

To screen the presence of somatic mutations, RT-PCR-SSCP analysis of *Cav-1* and *Cav-2* was performed using 3 sets of primers that were designed to cover the entire coding region of the genes. Twenty μl of PCR products mixed with 10 μl of 0.5 N NaOH, 10 mM EDTA, and 15 μl of denaturing loading buffer (95% formamide, 20 mM EDTA, 0.05% bromophenol blue and 0.15% xylene cyanol). After heating at 95 °C for 5 min, samples were loaded in wells pre-cooled to 4 °C and run using 8% nondenaturing acrylamide gels containing 10% glycerol at 4–8 °C and 18–22 °C.

### 5-Aza-dC treatment and bisulfite DNA sequencing analysis

To assess re-activation of *Cav-1* expression, cells were treated with 5 μM of 5-Aza-dC (Sigma, St. Louis, MO) for 4 days. For bisulfite sequencing analysis, 1 μg of genomic DNA was incubated with 3 M sodium bisulfite (pH 5.0) and DNA samples were purified as described previously [20]. Fifty ng of bisulfite-modified DNA were subjected to PCR amplification of the 37 CpG sites within the promoter and exon 1 using primers P1/P2 (Table 1). The PCR products were cloned into pCR^II^ vectors (Invitrogen Corporation, Alemeda, CA) and 5 clones of each specimen were subjected to DNA sequencing analysis to determine the methylation status.

### Immunoblotting assay

Cells were lysed in a lysis buffer containing 60 mM octylglucoside, 20 mM Tris (pH 7.4), 150 mM NaCl, 1 mM EDTA, 1 mM EGTA, 1% Triton X-100, 2.5 mM sodium phosphate, 1 mM β-glycerophosphate, 1 mM Na_3_VO_4_, 1 μg/ml leupeptin and 1 mM PMSF. Twenty μg of total protein were supplemented with Laemmli buffer and loaded on a 10% SDS-polyacrylamide gel for electrophoresis. Western analyses were performed using antibodies specific for Cav-1 (sc-894, Santa Cruz, CA), Cav-2 (610,685, BD bioscience, CA), EGFR (#4267, Cell Signaling, Danvers, MA), RAF (#2330, Cell Signaling), MEK1/2 (#9911, Cell Signaling), ERK (#9101, Cell Signaling), AKT (#4060, Cell Signaling), JNK (#4668, Cell Signaling), and β-tubulin (T8328, Sigma). Antibody binding was detected by enhanced chemiluminescence (Amersham Biosciences, Piscataway, NJ) using a secondary antibody conjugated to horseradish peroxidase.

### Immunofluorescence and immunohistochemistry (IHC) assay

For immunofluorescence assay, cells were fixed with 4% formaldehyde, permeabilized with 0.2% Triton X-100 and blocked with 2% bovine serum albumin-PBS. Slides were incubated with anti-GFP antibody and fluorescent imaging was obtained with a confocal laser scanning microscope (Carl Zeiss, Jena, Germany). IHC study was carried out using tissue arrays (SuperBioChips Laboratory, Seoul, Korea) and Vectastain ABC (avidin-biotin-peroxidase) kit (Vector Laboratories) as described previously [21]. Briefly, slides were incubated with Cav-1 antibody overnight using biotin-free polymeric horseradish peroxidase-linker antibody conjugate system. Slides were counterstained with hematoxylin, dehydrated and visualized using an Olympus CK40 microscopy (Tokyo, Japan). For the immunoreactive score, we established a 1- to 12-point system by multiplying the percentage of positive cells by the intensity of the staining score. Two pathologists performed the assessment of immunostaining sections. Immunoreactive scores of 0–5 were classified as negative, and scores of 6–12 were regarded as positive [22].

### Ras activity assay

Cells were lysed with Mg-containing lysis buffer containing 25 mM HEPES (pH 7.5), 150 mM NaCl, 1% Igeal CA-630, 10% Glycerol, 25 mM NaF, 10 mM MgCl_2_, 1 mM EDTA, 1 mM Sodium orthovanadate, 10 μg/ml Leupeptin, 10 μg/ml Aprotinin, and 1 mM PMSF. Cell lysates were mixed with RAF-1 RBD agarose (Millipore, Billerica, MA) and the reaction mixture were rocked gently at 4 °C for 30 min. Agarose beads were collected by centrifugation, washed 3 times with lysis buffer, and resuspended in 2X Laemmli sample buffer. Samples were electrophoresed on SDS-PAGE and immunoblotted.

### Expression plasmids, siRNA, shRNA, and transfection

GFP or Flag-tagged *Cav-1* gene was cloned into the pcDNA3.1-V5-His (Invitrogen Corporation) and the pEGFP-N3 vector (Clontech, Mennheim Germany) using the Expand High Fidelity PCR system (Roche Molecular Biochemicals, Palo Alto, CA). siRNA against Cav-1 (5′-AACCAGAAGGGACACACAGUU-3′) and ERK2 (5′-CACCAUUCAAGUUCGACAUUU-3′) were synthesized by Dharmacon Research (Lafayette, CO). shRNA plasmid for Cav-1 (5′-caccACCTTCACTGTGACGAAATACTGGTTtctcAACCAGTATTTCGTCACAGTGAAGG-3′) was constructed by Genolution (Seoul, Korea). Transfection was performed using FuGENE 6 (Roche Molecular Biochemicals) or Oligofectamine (Invitrogen Corporation).

### Cell proliferation, DNA synthesis, and colony formation assay

To measure in vitro cellular growth, cells were seeded at the density of 4 × 10^4^ cells per well in triplicate and cell numbers were counted using a hemocytometer at 24 h intervals. For flow cytometry analysis, cells were fixed with 70% ethanol and resuspended in PBS containing 50 mg/ml RNase and 50 mg/ml propidium iodide (Sigma). The assay was performed on a FACScan flow cytometer (Becton Dickinson, San Jose, CA) and analyzed using Modfit software (Becton Dickinson). For DNA synthesis assay, cells were pulse-labeled for 4 h with 1 μCi/ml [^3^H] thymidine and harvested with lysis buffer (0.1 N NaOH, 1% SDS). The cell lysates were mixed with the liquid scintillation cocktail (ICN Inc., Irvine, CA) and [^3^H]thymidine incorporation was counted with Scintillation Counter (Wallac, Milton Keynes, UK). For colony formation assay, 1 × 10^5^ cells per dish were maintained in the presence of G418 (1600 μg/ml) for 4–6 weeks. Selection medium were replaced every 2 days. Colonies were fixed with methanol for 15 min and stained with 0.05% crystal violet in 20% ethanol.

### Statistical analysis

The results of cell growth, apoptosis and colony forming assays were expressed as mean ± SD. A student’s t-test was used to determine the statistical significance of the difference. The Chi-square test was used to determine the statistical significance of expression and methylation levels between tumor and normal tissues. A *P* value of less than 0.05 was considered significant.

## Results

### Cav-1 expression is commonly down- or up-regulated in gastric cancers

**Fig. 1:**
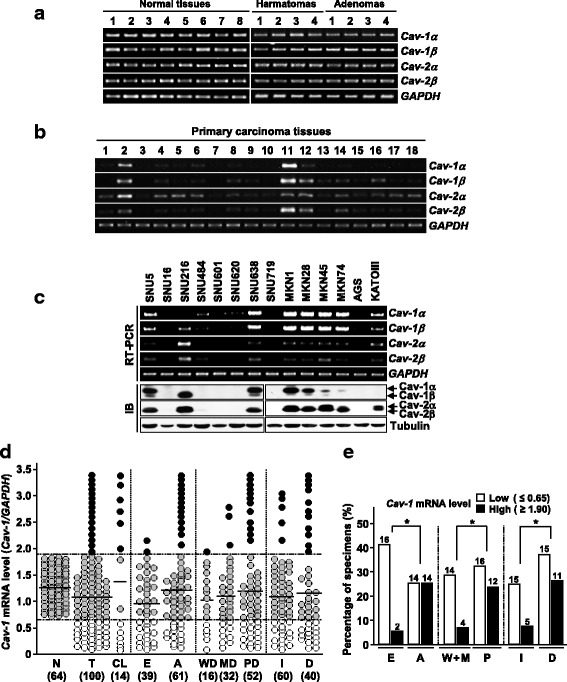
Expression status of Cav-1 in gastric tissues and cell lines. **a** Quantitative RT-PCR analysis of caveolin expression in normal and benign tumor tissues. **b, c** Caveolin expression in gastric tissues and cell lines. IB, immunoblot. **d, e** Association of *Cav-1* expression levels with tumor stages, grades, and types. N, normal tissues; T, primary tumors; CL, cell lines; E, early; A, advanced; WD; well-differentiated; MD, moderately differentiated; PD, poorly differentiated; I, intestinal; D, diffused. Bar indicates the mean levels of each specimen group

To explore the implication of caveolin alteration in gastric tumorigenesis, we initially investigated its mRNA expression status in 14 cancer cell lines and 180 gastric tissues, including 100 matched sets of primary tumors and adjacent noncancerous tissues. While all 64 normal and 16 benign tumor tissues we examined showed easily detectable mRNA levels of α and β isoforms of *Cav-1* and *-2*, a substantial fraction of cell lines and primary tumors displayed abnormally low or high expression of the transcripts (Fig. 1a-c). An immunoblot assay revealed that both Cav-1 and -2 protein levels are well consistent with their mRNA levels, indicating that caveolin expression is controlled mainly at the transcript level (Fig. 1c). However, MKN45 and MKN74 cells showed relatively low Cav-1 protein levels compared to their mRNA levels. Except for SNU216 cells which show no *Cav-1α* but high *Cav-1β* expression, all cell lines and tissue specimens we examined displayed comparable expression patterns of the isoforms (Fig. 1c). *Cav-1* expression levels (*Cav-1/GAPDH*) were detected in a range of 0.66–1.89 (mean 1.27), 0.07–3.43 (mean 1.09), and 0.00–3.40 (mean 1.41) in normal tissues, primary tumors, and cancer cell lines, respectively (Fig. 1d). Based on expression ranges of normal tissues, 30 (30%) and 16 (16%) of 100 primary tumors were classified as abnormally low and high *Cav-1* expressors, respectively and 6 (42.9%) and 6 (42.9%) of 14 cancer cell lines were classified as abnormally low and high expressors, respectively. Abnormal reduction of *Cav-1* was significantly more frequent in early (16 of 39, 41%) versus advanced tumors (14 of 61, 23%) whereas abnormal elevation was more frequent in advanced (14 of 61, 23%) versus early (2 of 39, 5.1%) and high (12 of 52, 23.1%) versus low (4 of 48, 8.3%) grade tumors (Fig. 1e). Meanwhile, both abnormal reduction and elevation of *Cav-1* were more frequently observed in diffuse versus intestinal type tumors.

**Fig. 2:**
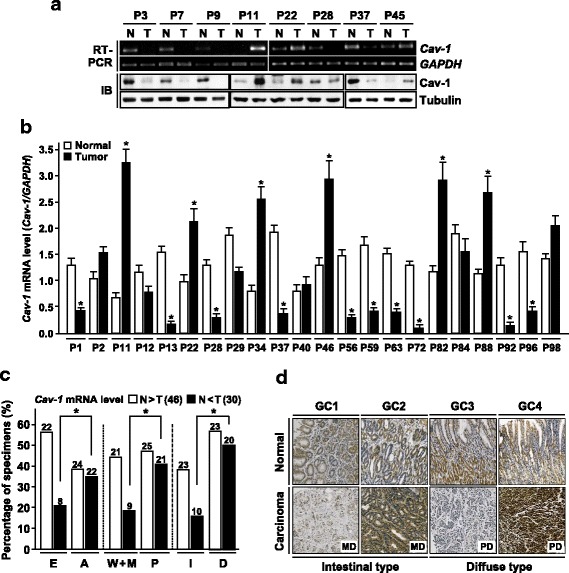
Comparison of Cav-1 expression in matched tissue sets. **a** RT-PCR and immunoblot analysis of Cav-1 expression in cancer and adjacent noncancerous tissues obtained from the same cancer patients. P, patient. **b** Relative *Cav-1* mRNA levels in cancer and adjacent noncancerous tissues. **c** Comparison of Cav-1 expression in matched sets and its association with tumor stages, grades and types. P, patient. **d** Immunohistochemical analysis of Cav-1 in tumor and matched normal tissues. GC, gastric cancer

Next we compared Cav-1 expression in normal and tumor tissues obtained from 100 cancer patients. Compared to adjacent noncancerous tissues, 46 and 30 cancers tissues showed down-and up-regulation of *Cav-1* mRNA, respectively (Fig. 2a, b). An immunoblot assay for eight representative tissue sets showed that mRNA levels analyzed by RT-PCR correlate well with protein levels in both normal and cancer tissues (Fig. 2a). *Cav-1* reduction in cancerous lesion was more frequent in early (22 of 39, 56.4%) versus advanced (24 of 61, 39.3) tumors while its elevation was more common in advanced (22 of 61, 36.1%) versus early (8 of 39, 20.5%) tumors, further supporting the biphasic alteration of *Cav-1* expression during gastric tumor progression (Fig. 2c). To further confirm the finding, we performed an IHC study using additional 40 matched tissue sets. As predicted, a substantial decrease and increase in Cav-1 immunopositivity were observed in 19 (47.5%) and 11 (27.5%) cancer tissues, respectively (Fig. 2d). Collectively, these results indicate that *Cav-1* is commonly down- and up-regulated in early and advanced gastric cancers, respectively, suggesting the oncogenic conversion of its function during tumor progression.

### Bidirectional alteration of Cav-1 is associated with promoter hyper- and hypo-methylation

**Fig. 3:**
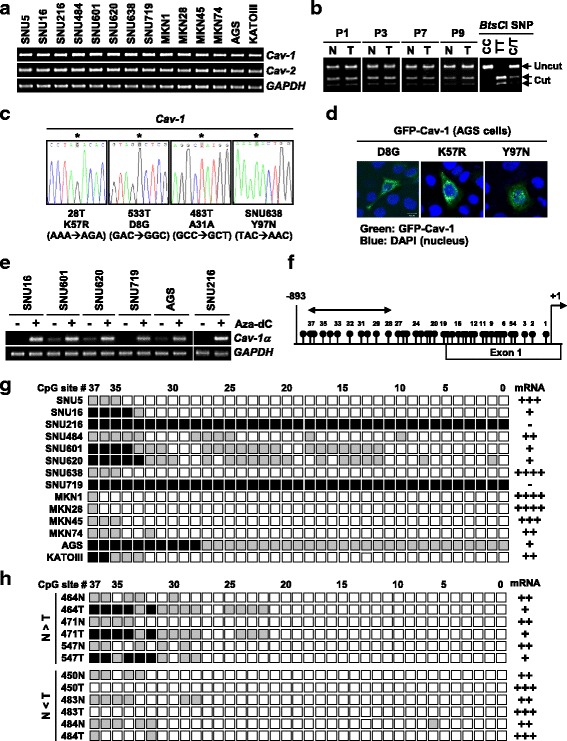
Mutation and promoter methylation analysis of Cav-1 in gastric cancers. **a** Genomic levels of *Cav-1* in 14 cancer cell lines. **b** LOH analysis of the *Cav-1* gene. Exon 3 region containing a SNP (5′-AGCATC*C*/*T*-3′) was amplified by PCR and digested with endonuclease *BtsC*I. P, patient; N, normal; T, tumor. **c** Sequence alterations of *Cav-1* in cell lines and tumors. **d** Immunofluorescence assay for expression and subcellular localization of mutant *Cav-1* proteins in AGS cells. **e** Quantitative RT-PCR analysis showing re-activation of *Cav-1* expression after 5-Aza-dC treatment. **f** A map of the 37 CpG sites in the promoter and exon 1 region of *Cav-1*. The first nucleotide of ATG start codon is indicated by an arrow at +1. **g** Methylation status of the 37 CpGs in 14 cell lines. The gene region comprised of 37 CpGs was amplified by PCR and the PCR products were cloned. Five plasmid clones were sequenced for each cell line. Black, gray, and white circles represent complete methylation (4–5 clones), partial methylation (1–3 clones), and unmethylation, respectively. **h** Methylation status of the CpGs in primary tumors. N, adjacent noncancerous tissue; T, tumor tissue

To define whether altered expression of *Cav-1* in cancers is caused by gene deletion or amplification, genomic status of *Cav-1* was analyzed. Semi-quantitative DNA-PCR assay revealed that all cancer cell lines and primary tumors we tested have *Cav-1* gene levels comparable to those of normal cells (Fig. 3a). An allelotyping assay of 100 matched sets using an intragenic single nucleotide polymorphism (5′-AGCATC*C*/*T*-3′) in exon 3 and the endonuclease *BtsC*I digestion identified 49 informative cases, but none of these showed detectable allelic imbalance between normal and cancer tissues (Fig. 3b). Interestingly, however, PCR-SSCP analysis of 14 cell lines and 50 tumors detected 3 missence and 1 silent sequence alterations in the *Cav-1* gene from SNU638 cells (Y97N, TAC to AAC) and three primary tumors (K57R, AAA to AGA; D8G, GAC to GGC; A31A, GCC to GCT) (Fig. 3c). An immunofluorescence microscopic assay revealed that all of these mutant Cav-1 proteins exhibited both perinuclear and plasma membrane localization with a punctuated manner (Fig. 3d).

Next, we tested whether differential expression of *Cav-1* is due to the epigenetic alteration of transcription. In 6 cell lines with no or low expression, *Cav-1* transcript level was markedly increased after treatment with the demethylating agent 5-Aza-dC, suggesting that abnormal *Cav-1* down-regulation in these cells might be associated with promoter hypermethylation (Fig. 3e). On this basis, we performed bisulfite DNA sequencing analysis of 37 CpG sites within the promoter and exon 1 region (Fig. 3f). Five PCR clones from each specimen were sequenced to determine methylation frequency at individual CpG sites. Among 37 CpGs, 21–37 sites were partially or completely methylated in 6 cell lines with no or low *Cav-1* expression, whereas only 1–4 sites showed methylation in 6 cell lines with high *Cav-1* expression (Fig. 3g). In particular, methylation status of 10 CpGs (numbered 28–37 in Fig. 3f) within nucleotides −446 to −772 was most tightly associated with mRNA expression status. While all of these 10 CpGs were completely methylated in 3 cell lines (SNU216, SNU719, and AGS) with extremely low expression, partial methylation only at 1–4 sites was found in 6 high expressor cell lines. We next compared methylation status in tumors and adjacent noncancerous tissues using 5 low *Cav-1* and 5 high *Cav-1* tumors. Low and high *Cav-1* tumors showed methylation at 9–14 and 0–2 sites, respectively while noncancerous tissues showed 4–6 sites methylation (Fig. 3h). Consistent with cell lines, methylation at the 10 distant CpG sites showed a tight association with mRNA expression status, supporting that methylation status of this region is crucial for the transcriptional control of the gene. These results indicate that aberrant promoter hyper- or hypo-methylation of *Cav-1* is a common event in gastric tumorigenesis and tightly correlates with its bidirectional expression.

### Cav-1 exerts opposite effects on growth of low- and high-expressing tumor cells

**Fig. 4:**
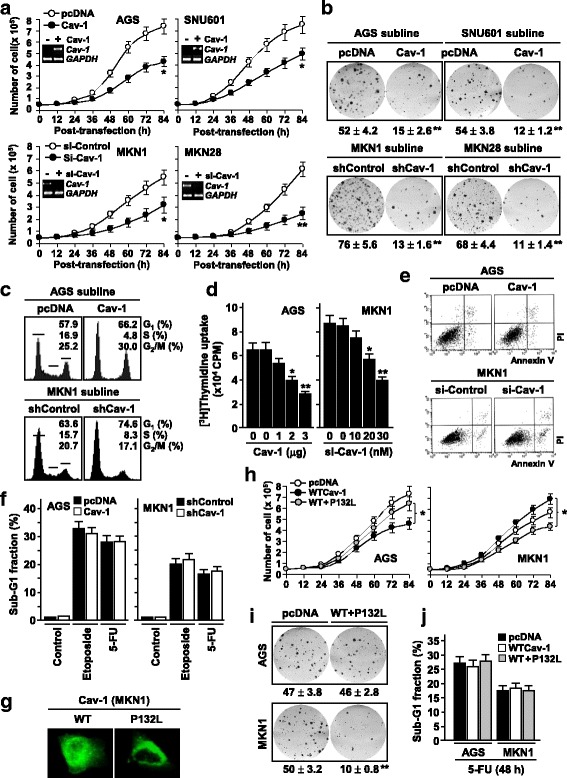
Cav-1 effect on tumor cell growth. **a** Opposite effects of Cav-1 on growth of low-and high-expressing tumor cells. AGS and SNU601 were transfected with 2 μg of WT-Cav-1 expression or empty vector (pcDNA), and MKN1 and MKN28 were transfected with 30 nM of si-Cav-1 or si-Control. Data represent means of triplicate assays (Bars, SD) (*, *P* < 0.05; **, *P* < 0.01). **b** Cav-1 effect on colony forming ability of tumor cells. Cav-1-expressing sublines of AGS and SNU601 and Cav-1-depleted sublines of MKN1 and MKN28 were maintained in the presence of G418 (1600 μg) for 4–6 weeks. **c** Flow cytometric analysis of Cav-1 effect on cell cycle progression. **d** [^3^H]thymidine uptake assay showing opposite effects of Cav-1 on DNA synthesis in AGS and MKN1 cells. **e** Flow cytometric analysis of Annexin V-positive cells showing no significant effect of Cav-1 on apoptosis. **f** Flow cytometric analysis of sub-G1 fraction. Cells were exposed to etoposide (15 μM) or 5-FU (15 μM) for 48 h. **g** Subcellular distribution of WT- and MT-Cav-1 (P132L) proteins. (**h**-**j**) Effect of a dominant negative Cav-1 mutant (P132L) on tumor cell growth, colony formation, and apoptosis

To address the biological significance of bidirectional alteration of Cav-1 in gastric tumor progression, we examined its effects on tumor cell growth. Ectopic overexpression of wild-type (WT) Cav-1 in AGS and SNU601 cells (low *Cav-1*) resulted in 36–42% inhibition of cellular growth, whereas siRNA-mediated depletion of endogenously expressed Cav-1 in MKN1 and KNK28 cells (high *Cav-1*) caused 41–60% reduction in cellular growth (Fig. 4a), indicating that Cav-1 has opposite roles in the growth regulation of low and high expressor cells. Consistently, Cav-1-expressing stable sublines of AGS and SNU601 showed 71–78% decrease in colony formation whereas shCav-1 sublines of MKN1 and MKN28 showed 83–85% decrease in colony formation (Fig. 4b). Flow cytometric analysis of the cell cycle showed that AGS/Cav-1 and MKN1/shCav-1 sublines showed higher fractions of the G1 phase cells compared to control sublines (Fig. 4c). [^3^H]Thymidine uptake assay also revealed that DNA synthesis is inhibited by Cav-1 expression in AGS cells but by its depletion in MKN1 cells (Fig. 4d). However, Cav-1 did not affect both basal and genotoxic stress-induced apoptosis (Fig. 4e, f). Next, we utilized a dominant negative mutant form of Cav-1 (P132L) to further test the opposite effects of Cav-1 in these cells [23]. As reported, Cav-1/P132L proteins were localized predominantly in the perinuclear region, indicating the loss of its caveolae-scaffolding protein (Fig. 4g). Both the growth-inhibiting (AGS) and growth-promoting (MKN1) effects of Cav-1 were significantly suppressed when Cav-1/P132L was co-transfected while apoptotic response to 5-FU was not affected by Cav-1/P132L (Fig. 4h-j). Collectively, these findings indicate that up- and down-regulated *Cav-1* in gastric cancers has opposite roles in the regulation of tumor cell growth.

### Opposite functions of Cav-1 stems from reciprocal regulation of ERK phosphorylation

**Fig. 5:**
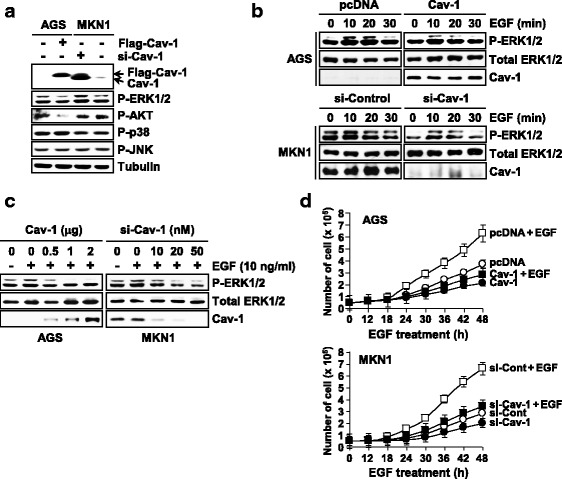
Cav-1 regulation of ERK signaling. **a** Opposite effects of Cav-1 on ERK1/2 phosphorylation. AGS and MKN1 cells were transfected with WT-Cav-1 or si-Cav-1 and its effect on phosphorylation levels of AKT, ERK1/2, p38, and JNK were examined by an immunoblot assay. **b** Effect of Cav-1 on EGF-induced phosphorylation of ERK1/2. Cells transfected with WT-Cav-1 or si-Cav-1 were exposed to EGF (10 ng/ml) and phopho-ERK1/2 level was determined. **c** A dose-associated effect of Cav-1 on EGF-induced ERK1/2 phosphorylation. **d** Opposite effects of Cav-1 on EGF-induced cell growth. WT-Cav-1- or si-Cav-1-transfected cells were incubated with EGF (10 ng/ml). Data represent means of triplicate assays (Bars, SD)

To address the molecular basis for the opposite roles for Cav-1 in the regulation of tumor cell proliferation, we initially defined its effect on growth-regulating signaling components. As shown in Fig. 5a, phospho-p38 and phospho-JNK levels were not affected by Cav-1 in both AGS and MKN1 cells while phospho-AKT level was greatly decreased by Cav-1 expression in AGS but not affected by Cav-1 depletion in MKN1 cells. This result suggests that Cav-1 down-regulation but not its up-regulation is associated with AKT activation in gastric tumor cells. Intriguingly, we found that phospho-ERK1/2 level is decreased by Cav-1 expression in AGS cells and by Cav-1 depletion in MKN1 cells (Fig. 5a, b). Likewise, EGF-induced ERK1/2 phosphorylation was blocked by Cav-1 expression in AGS cells but by Cav-1 depletion in MKN1 cells in a dose-associated manner (Fig. 5c). Consistently, the mitogenic effect of EGF was profoundly attenuated by Cav-1 expression and depletion in AGS and MKN1 cells, respectively (Fig. 5d). These support that opposite effects of Cav-1 on cell growth are associated with its reciprocal regulation of ERK signaling.

### Cav-1 evokes opposite effects on RAF inhibitory phosphorylation in low- and high-expressing cells

**Fig. 6:**
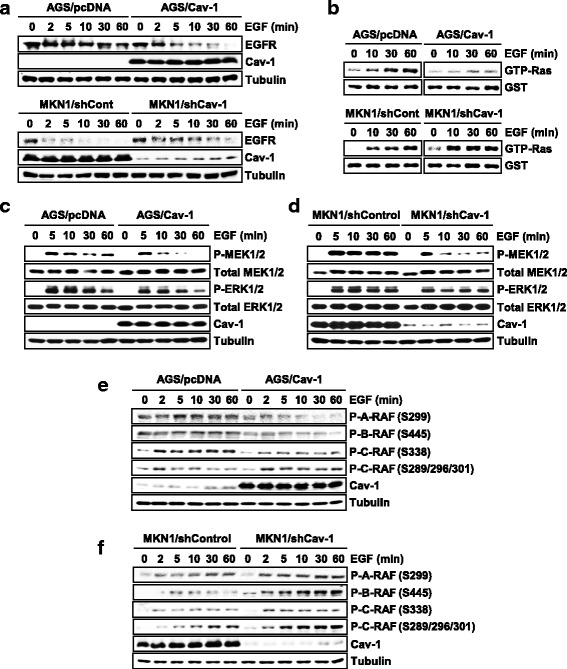
Opposite effects of low and high Cav-1 on inhibitory phosphorylation of RAF. **a** Cav-1 inhibition of EGFR stability. WT-Cav-1- or shCav-1-expressing cells were exposed to EGF (10 ng/ml) and EGFR level was determined using an immunoblot assay. **b** Effect of Cav-1 on Ras activity. GTP-Ras levels were measured using Ras activity assay. **c**, **d** Opposite effects of Cav-1 on EGF-induced MEK1/2 phosphorylation. **e**, **f** Effect of Cav-1 on EGF-induced RAF phosphorylation. Stimulatory phosphorylation of A-RAF (P-S299), B-RAF (P-S445) and C-RAF (P-S339) and inhibitory phosphorylation of C-RAF (P-S289/296/301) were detected using antibodies specific to phospho-RAF isoforms

To further dissect the opposite roles for Cav-1 in ERK signaling, we compared its regulation of the EGFR-Ras-RAF-MEK-ERK signaling cascade in AGS and MKN1 cells. Upon ligand stimulation, EGFR is internalized and its signaling is attenuated by ubiquitin-mediated degradation [24]. Compared with controls, Cav-1-transfected AGS cells showed more rapid degradation of EGFR whereas siCav-1-transfected MKN1 cells exhibited more delayed degradation, indicating that EGFR degradation is promoted by Cav-1 in both cells (Fig. 6a). A Ras kinase activity assay also revealed that Cav-1 represses Ras activity in both cells (Fig. 6b). However, EGF-induced phosphorylation of MEK1/2 was attenuated by Cav-1 in AGS cells but by siCav-1 in MKN1 cells, suggesting that kinase(s) upstream of MEK and downstream of Ras might be a target for the reciprocal regulation of Cav-1 (Fig. 6c, d).

The C-RAF kinase is a crucial molecule to transmit Ras signals to MEK and its activity is precisely controlled by interplays with isoforms (A-RAF and B-RAF) and differential phosphorylation at multiple sites. In particular, C-RAF has been known to be regulated by ERK1/2 via direct feedback phosphorylation [25]. Activated ERK1/2 phosphorylates C-RAF at multiple serine residues (S29, S289, S296, S301, and S642) and thus blocks the MEK-activating function of active C-RAF (P-S338). On this basis, we characterized Cav-1 effect on RAF isoforms and their phosphorylation status. In both AGS and MKN1 cells, Cav-1 was found to down-regulate active phosphorylation of three RAF isoforms (A-RAF/P-S299, B-RAF/P-S445, and C-RAF/P-S338) (Fig. 6e, f). Intriguingly, however, the inhibitory phospho-RAF (P-S289/S296/S301) was increased by Cav-1 expression in AGS cells but by Cav-1 depletion in MKN1 cells. Therefore, these results strongly suggest that ERK inhibition of C-RAF by feedback phosphorylation might be a critical target that is reciprocally regulated by Cav-1 in low- and high-expressor cancer cells.

### Overexpressed Cav-1 blocks the RAF-ERK negative feedback loop

**Fig. 7:**
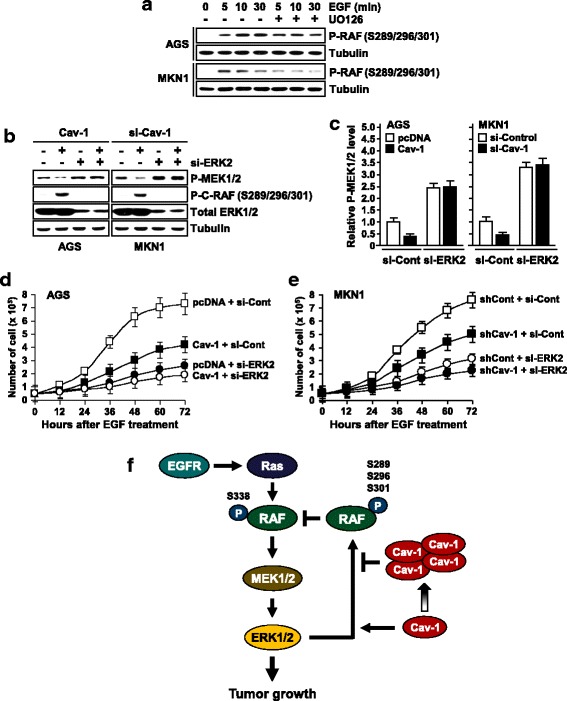
Reciprocal regulation of the RAF-ERK feedback loop by Cav-1. **a V**alidation of the RAF-ERK feedback regulation. Cells were treated with the MEK inhibitor UO126 and its effect on the inhibitory phosphorylation level of C-RAF was determined. **b**, **c** Effect of ERK depletion on Cav-1 regulation of RAF, MEK, and ERK. Cells were co-transfected with si-ERK2 and WT-Cav-1 or si-Cav-1. **d**, **e** Effect of ERK depletion on Cav-1 regulation of EGF-induced cell growth. WT-Cav-1- or shCav-1-expressing sublines were transfected with si-ERK2 and incubated with EGF (10 ng/ml). Data represent means of triplicate assays (Bars, SD). **f** Schematic representation of the opposite functions of Cav-1 in the regulation of the RAF-ERK feedback phosphorylation loop in gastric cancer cells

To elicit whether the opposite effects of Cav-1 on cell growth is associated with its reciprocal regulation of the ERK-RAF feedback loop, we tested Cav-1 effect on inhibitory phosphorylation of RAF under ERK-blocked conditions. EGF treatment induced inhibitory phosphorylation of RAF (P-S289/S296/S301) in both AGS and MKN1 cells and this effect was attenuated by the MEK inhibitor UO126, supporting the ERK inhibition of RAF by feedback phosphorylation (Fig. 7a). We next tested whether the reciprocal control of MEK1/2 phosphorylation by Cav-1 is abolished if ERK is depleted. ERK depletion led to an increase in phospho-MEK1/2 level, possibly due to increased RAF kinase activity by loss of the RAF-ERK feedback loop (Fig. 7b, c). As predicted, down-regulation of MEK1/2 phosphorylation by Cav-1 expression in AGS cells and by its depletion in MKN1 cells was not detected when ERK2 expression is knock-downed (Fig. 7b). Furthermore, the opposite effects of Cav-1 on EGF-induced cell proliferation were substantially debilitated in ERK-depleted cells (Fig. 7d, e). Collectively, these results indicate that the bidirectional alteration of Cav-1 expression in gastric cancers is linked to its opposite effects on tumor cell proliferation, which stems from the reciprocal control of the RAF-ERK negative feedback loop (Fig. 7f).

## Discussion

Cav-1 has opposite functions in tumorigenesis depending on the cellular contexts [26]. However, the molecular mechanism underlying the differential effects of Cav-1 on tumor growth has been poorly defined. In the present study, we observed a bidirectional alteration of Cav-1 expression in gastric cancers, which is linked to the mitogenic conversion of its function. Our study provides evidence that the mitogenic conversion of Cav-1 function is associated with the switch of its role for the RAF-ERK feedback phosphorylation loop.

Previous IHC studies reported contrasting results on Cav-1 expression in gastric cancers [27–30]. A study using frozen tissues and a monoclonal antibody revealed that Cav-1 is expressed in only a small fraction of intestinal type cancers [28]. Meanwhile, a study using formalin-fixed specimens and a polyclonal antibody showed that Cav-1 is expressed in both diffuse and intestinal types at variable levels [30]. In the present study, we identified that both intestinal and diffuse types of cancers express Cav-1 at highly variable levels. However, Cav-1 down-regulation was significantly more frequent in early versus advanced tumors while its up-regulation was more common in advanced versus early tumors and high versus low grade tumors, supporting that alteration of Cav-1 expression is associated with the oncogenic switch of its function [29, 31]. Our data thus suggest that Cav-1 may act as a stage-specific growth modulator in gastric cancer, which is inactivated during the early stages of tumorigenesis and its subsequent elevation confers growth advantages and malignant progression [26, 32].

It is becoming clear that altered expression of Cav-1 in tumor stroma, particularly in cancer-associated fibroblasts (CAFs), is linked to the malignant progression of various types of human cancers [33, 34]. A study showed that loss of CAFs Cav-1 promotes tumor microenvironment remodeling and tumor development [35]. However it was also reported that stromal Cav-1 favors tumor invasion and metastasis [36]. Therefore, the role for CAFs Cav-1 in tumorigenesis remains largely undefined. It was demonstrated that Cav-1 is not expressed in the epithelial compartment in normal gastric mucosa and in the metaplastic intestinal epithelium while its expression is significantly higher in advanced versus early cancers and an independent prognostic factor of poor survival [29]. In contrast, a recent study using quantum dots immunofluorescence histochemistry identified that epithelial Cav-1 expression gradually decreases with the progression of gastric cancer [37]. Interestingly, this study also showed that low Cav-1 expression in CAFs rather than in tumor cells predicts recurrence and survival in cancer patients, suggesting that loss of stromal Cav-1 heralds poor prognosis of gastric cancer patients, which is consistent with the finding in breast and prostate cancer [38, 39]. Although we did not characterize CAFs Cav-1 expression status in the current study, it was recognized that compared to normal gastric mucosa, noncancerous tissues adjacent to cancerous tissues exhibit much variable levels of Cav-1 mRNA. Further studies will be required to address whether Cav-1 gene expression in CAFs also shows a bidirectional alteration due to promoter hypo- and hyper-methylation during gastric tumor progression.

Mutational alteration of the *Cav-1* gene has been rarely found in human cancers. However, *Cav-1* mutations were reported in certain tumor types [8]. A mutant Cav-1 (P132L) found in scirrhous breast cancer was identified to exert a dominant negative function by cytoplasmic retention [8]. Interestingly, we detected three missence and one silent sequence alterations in *Cav-1* from 3 of 50 primary tumors and 1 of 14 cancer cell lines. Our preliminary data suggest that all of these mutants are expressed. The central α-helical region of Cav-1 protein interacts with the catalytic subunit of protein kinase A (PKAcat) through a hydrogen bond between its Y97 residue and W196 residue of PKAcat [38]. Our finding of Y97N mutation in the SNU638 cancer cell line thus raises the possibility that Cav-1 regulation of PKA signaling might be altered in these gastric cancer cells.

*Cav-1* null mice exhibit increases in tumor incidence, tumor area, and tumor number compared with wild-type counterparts [39]. In contrast, prostate cancer cells secrete Cav-1, which stimulates clonal growth of tumor cells, and high Cav-1 expression exerts anti-apoptotic effect under clinically relevant circumstances [40, 41]. Our previous study showed that high Cav-1 expression enhances the metastatic potential of gastric tumor cells by increasing the adhesion ability of the cells to endothelium through the regulation of cell surface VCAM [42]. In the present study, we observed that Cav-1 provokes either a growth-inhibiting or growth-promoting effect in gastric cancers, and this property of Cav-1 is associated with its reciprocal regulation of ERK. Signaling components, including Ras, RAF, MEK, and ERK, are known to be compartmentalized within caveolin-rich membrane domains [2]. However, there is a disagreement in the data concerning whether Cav-1 plays an inhibitory or stimulatory role in Ras-ERK signaling in cancer cells. We found that Cav-1 reciprocally regulates MEK and ERK in low- and high-expressing tumor cells. RAF is a crucial factor to transmit growth factor-induced Ras signals to MEK, and its activity is precisely regulated by differential phosphorylation of RAF isoforms and a negative feedback loop between RAF and ERK [25]. We found that Cav-1 evokes opposite effects on the inhibitory phosphorylation of RAF in low- and high-expressing cells through the reciprocal control for the ERK-mediated inhibitory phosphorylation of RAF. We validated that in EGF-treated gastric tumor cells, RAF is inhibited by ERK feedback phosphorylation and this feedback loop is differentially regulated by Cav-1 in these two cell types. The opposite effects of Cav-1 on tumor cell growth were disrupted if ERK expression is depleted, supporting that the mitogenic conversion of Cav-1 effect is linked to its reciprocal regulation of the ERK feedback phosphorylation of RAF. Although further studies are required to understand the molecular mechanism underlying the Cav-1 regulation of the RAF-ERK negative feedback loop, studies suggest that Cav-1 may affect ERK feedback regulation of signaling components, including RAF, MEK, and KSR1 [15, 43, 44]. It is thus conceivable that MEK-ERK signaling is activated through Cav-1 up- and down-regulation in early and advanced tumors, respectively.

## Conclusions

Cav-1 acts as a positive or negative regulator of tumor cell growth via the reciprocal control for the RAF-ERK feedback loop, and the mitogenic switch of Cav-1 function is tightly linked to bidirectional alteration of its expression in tumor progression. Therefore, Cav-1 represents one critical modulator of the RAF-ERK negative feedback loop, adding a new mechanism by which Cav-1 functions as a regulator of tumor growth.

## Abbreviations

EGF: epidermal growth factor
ERK: extracellular signal-related kinase
GAPDH: glyceraldehyde-3-phosphate dehydrogenase
JNK: c-Jun N-terminal kinase
MAPK: mitogen-activated protein kinase
MEK1: MAPK/ERK kinase 1
RT-PCR: reverse transcription-polymerase chain reaction
